# 49824 Determining factors that influence adoption of new post-stroke physical rehabilitation devices

**DOI:** 10.1017/cts.2021.549

**Published:** 2021-03-30

**Authors:** Corey Morrow, Emily Johnson, Kit Simpson, Na Jin Seo

**Affiliations:** 1 Medical University of South Carolina; 2 Medical University of South Carolina and Ralph H. Johnson VA Medical Center

## Abstract

ABSTRACT IMPACT: This work will accelerate the translation of post stroke rehabilitation devices from the research lab to clinic use. OBJECTIVES/GOALS: Rehabilitation device efficacy alone does not lead to adoption into clinical practice. The objective of this work was to increase understanding of the landscape for clinical adoption of post-stroke physical rehabilitation devices. METHODS/STUDY POPULATION: We conducted interviews with 107 stakeholders including patients who have had strokes, rehab directors, and physical/occupational therapists to understand their viewpoints for adopting new rehabilitation devices. To contribute to previous literature, interviews were analyzed qualitatively using direct content analysis to provide more specific details about the most appropriate adoption settings, specific roles for stakeholders, and drivers for all stakeholders involved in the adoption process. RESULTS/ANTICIPATED RESULTS: Unique to this work, care settings in which therapy goals are best aligned for restorative devices were found to be outpatient rehabilitation, followed by inpatient rehabilitation. Therapists are the major influencers for adoption because they typically introduce new rehabilitation devices to patients for both clinic and home use. We also learned therapists’ utilization rate of a rehabilitation device influences a rehabilitation director’s decision to acquire the device for facility use. Additionally, device setup in <7 minutes will allow for increased use without reducing therapist productivity. DISCUSSION/SIGNIFICANCE OF FINDINGS: Rehabilitation device development should consider the best settings to first introduce the device, roles of each stakeholder, and drivers that influence each stakeholder to accelerate successful adoption of the developed device.

